# Storing and retrieving multiple images in 3D nonlinear photonic crystals

**DOI:** 10.1038/s41377-021-00631-5

**Published:** 2021-09-29

**Authors:** Ady Arie

**Affiliations:** School of Electrical Engineering, Fleischman Faculty of Engineering, Tel Aviv University, Tel Aviv, 69978 Israel

**Keywords:** Optics and photonics, Nonlinear optics

## Abstract

A nonlinear hologram enables to record the amplitude and phase of a waveform by spatially modulating the second order nonlinear coefficient, so that when a pump laser illuminates it, this waveform is reconstructed at the second harmonic frequency. The concept was now extended to enable the generation of multiple waveforms from a single hologram, with potential applications in high density storage, quantum optics, and optical microscopy.

## Quasi phase matching and nonlinear photonic crystals

Three wave mixing processes in quadratic nonlinear crystals are traditionally used for the generation of coherent light at new wavelengths, that are often hard to reach by direct lasing. Specifically, up-conversion process such as second harmonic generation and sum frequency generation are used for generation of visible and ultraviolet radiation from red or near-infrared lasers, whereas down-conversion processes, such as difference frequency generation and optical parametric oscillation are used to convert near-infrared light into the mid-infrared. In order to achieve an efficient process, it is necessary to conserve energy and momentum between the interacting photons. Let us consider for example the case of second harmonic generation, where two identical pump photons, each having energy $\hbar \omega _1$ and momentum $\hbar k_1$, are converted into a new photon with energy $\hbar \omega _2$ and momentum $\hbar k_2$. Energy conservation dictates that $\omega _2 = 2\omega _1$ and momentum conservation, also known as phase matching, means that $k_2 = 2k_1 + {\Delta}k$. The last term, ${\Delta}k$, is the crystal quasi momentum, that originates from spatial modulation of the quadratic nonlinear coefficient of the crystal. The concept of modulating the nonlinear coefficient to satisfy momentum conservation was proposed by Nobel Laureate Nicolaas Bloembergen and co-workers in a seminal paper they published in 1962^1^, and is called quasi phase matching. It is important to emphasize that without this modulation, the momentum is not conserved in most cases, and the efficiency of the process is negligible. Quasi phase matching was first explored in the case of periodic one dimensional modulation with a period of $2\pi /{\Delta}k$, but in 1998 Vincent Berger^2^ proposed to modulate the nonlinear coefficient in two or three dimensions. He referred to these structures with modulated nonlinear susceptibilities as nonlinear photonic crystals, in analogy to the linear photonic crystals^3^, in which the linear susceptibility is a periodic function of space. The concept of nonlinear photonic crystal is not limited only to periodic modulation. Other modulation schemes, based on quasiperiodic^4,5^ or even random modulation^6^ were proposed and realized as well. These methods enable to simultaneously phase match several different processes, i.e., for different input pump frequencies or different propagation directions^7^.

## How to modulate the nonlinear coefficient?

The realization of nonlinear photonic crystals requires a method that will enable to modulate the nonlinear coefficient with micron-scale resolution. The main method used nowadays for that is electric field poling of ferroelectrics^8^: A patterned electrode with the desired modulation function is defined on one side of the crystal, usually by photolithography, and a planar electrode is sputtered on the other side. The ferroelectric crystal is mono-domain at start, i.e., its electrical dipoles are all oriented at the same direction. When a high voltage pulse is applied through the electrodes, it can invert the direction of the electric dipoles, throughout the entire thickness of the crystal, in the regions in which the patterned electrode was in contact with the crystal. This domain inversion also inverts the sign of the nonlinear coefficient. This method, therefore, enables binary modulation of the nonlinear coefficient, but it is based on planar configuration, hence it is only suitable for one-dimensional or two dimensional modulation patterns. For example, in LiNbO_3_ and LiTaO_3_, electric field poling enables to modulate the nonlinear coefficient in the plane of the X and Y crystallography axes, but it does not enable to modulate along the Z axis.

In 2018, two groups have demonstrated new methods for modulating the nonlinear coefficient in all three dimensions. These methods are based on tightly focusing a strong laser beam to selected points in the crystal. In one method, that was demonstrated for LiNbO_3_^9^, the laser locally heats the focal point so that the crystal becomes amorphous and the nonlinear coefficient is locally erased, whereas in the second method, that was demonstrated for barium calcium titanate^10^, the focusing creates a local temperature gradient which locally inverts the spontaneous polarization of the crystal, with corresponding inversion of the nonlinear coefficient.

## Nonlinear holography and nonlinear beam shaping

The ability to modulate the nonlinear coefficient in two or three dimensions^11^ opens new possibilities for nonlinear photonic crystals, in addition to frequency conversion. Specifically, it enables to borrow concepts that were originally developed in the field of computer generated holography, such as off-axis Fourier holography, and binary coding schemes based on the detour phase method^12^ and on discrete Fourier transform^13^ for shaping the spatial and spectral shape of the generated light in a nonlinear process. As a reminder, the hologram enables to record the amplitude and phase of a certain desired beam, so that when it is illuminated by a reference laser, this beam can be reconstructed. In a nonlinear hologram, the information is encoded by modulating the nonlinear coefficient, so that when a pump (reference) laser illuminates it, the desired beam is reconstructed at the second harmonic frequency. It is important to remember that one also needs to maintain phase matching in order to obtain an efficient generation of the desired beam.

These concepts were first employed, using electric-field poling, for the generation of beams that were structured in one transverse dimension, such as focused beam^14^, one-dimensional Airy beam^15^ and Hermite-Gaussian beams of the HG_0n_ family^16^. In that case, one of the crystal axis (the X axis) was employed for quasi phase matching while one transverse axis (the Y axis) was used for modulation. Later, another scheme was proposed, in which two transverse axes (X and Y) were used for modulation, and partial phase matching, based on the nonlinear Raman-Nath effect^17^, was employed. This enabled to nonlinearly generate and mix vortex beams^18^, HG_mn_ beams^19^, etc. However, the partial Raman-Nath scheme means that the conversion efficiency of these holograms is relatively low. With the recent introduction of laser-based 3D modulation^9,10^, this efficiency problem was finally resolved—the hologram can be designed to satisfy phase matching in the propagation direction, and to shape the beam in the two transverse directions^20,21^.

## Holographic multiplexing: storing and retrieving multiple images in a single nonlinear hologram

**Fig. 1:**
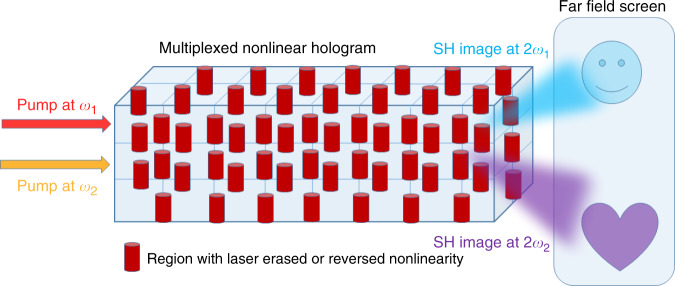
Retrieving two images from a multiplexed nonlinear hologram. The hologram is fabricated by erasing (or reversing the sign of) the nonlinearity in selected regions of a nonlinear crystal using a focused femtosecond laser. Each one of the two pump lasers will generate a different image on a far field screen

So far we assumed that there is only one pattern that we would like to store and retrieve in the nonlinear hologram. However, a nice feature of holography is the ability to store and retrieve multiple patterns in the same hologram, whereby each pattern is associated with its own reference beam, as illustrated in Fig. 1. In the recent paper of Chen et al.^22^, this concept was demonstrated. Two design examples were used—in one of them, three different images, of a heart, a moon, and musical note were stored, by 3D nonlinear modulation—achieved by local point by point laser erasing of the LiNbO_3_ nonlinear coefficient. The selection of the different patterns was achieved by tuning the pump frequency. The second harmonic process was phase matched each time only for one of these wavelengths. The authors have also shown another example of storing and retrieving six different patterns, each one consisting of a series of dots. The authors also estimate that with further improvements in the poling technology, up to 100 different patterns can be stored, provided that the pump can be tuned over 250 nm. In addition to the pump wavelength, other degrees of freedom such as the pump’s direction of propagation and the crystal temperature may enable to further increase the number of stored patterns.

## Potential applications

The ability to multiplex different patterns in a single nonlinear hologram may enable new and interesting applications. A straightforward application is high density storage of optical information in a compact single crystal. An added benefit is the security of the storage, since a specific reference beam is needed to retrieve a desired waveform. These nonlinear holograms may be useful for generation of shaped quantum light by spontaneous parametric down conversion^23^. Another potential application is in microscopy, where the short wavelengths that can be reached by second harmonic generation, can now be generated in a nonlinear hologram. This is useful for super resolved structured illumination microscopy, where the sample can be illuminated by different patterns that are generated by the multiplexed nonlinear hologram. Whereas the present demonstration^22^ was based on far field off-axis hologram, the multiplexing capability may also be relevant for other types of nonlinear holograms, that operate on axis and in the near field^24^. In order to enable efficient realizations of these exciting applications, it is still needed to increase the length and overall volume of the crystals that can be modulated using the laser poling method.

## References

[1] Armstrong JA (1962). Interactions between light waves in a nonlinear dielectric. Phys. Rev..

[2] Berger V (1998). Nonlinear photonic crystals. Phys. Rev. Lett..

[3] Joannopoulos, J. D. et al. *Photonic Crystals: Molding the Flow of Light*. 2nd ed. (Princeton: Princeton University Press, 2008).

[4] Zhu SN, Zhu YY, Ming NB (1997). Quasi-phase-matched third-harmonic generation in a quasi-periodic optical superlattice. Science.

[5] Lifshitz R, Arie A, Bahabad A (2005). Photonic quasicrystals for nonlinear optical frequency conversion. Phys. Rev. Lett..

[6] Baudrier-Raybaut M (2004). Random quasi-phase-matching in bulk polycrystalline isotropic nonlinear materials. Nature.

[7] Arie A, Voloch N (2010). Periodic, quasi-periodic, and random quadratic nonlinear photonic crystals. Laser Photonics Rev..

[8] Yamada M (1993). First-order quasi-phase matched LiNbO_3_ waveguide periodically poled by applying an external field for efficient blue second-harmonic generation. Appl. Phys. Lett..

[9] Wei DZ (2018). Experimental demonstration of a three-dimensional lithium niobate nonlinear photonic crystal. Nat. Photonics.

[10] Xu TX (2018). Three-dimensional nonlinear photonic crystal in ferroelectric barium calcium titanate. Nat. Photonics.

[11] Zhang Y (2021). Nonlinear photonic crystals: from 2D to 3D. Optica.

[12] Lohmann AW, Paris DP (1967). Binary Fraunhofer holograms, generated by computer. Appl. Opt..

[13] Lee WH (1979). Binary computer-generated hologram. Appl. Opt..

[14] Kurz JR (2002). Nonlinear physical optics with transversely patterned quasi-phase-matching gratings. IEEE J. Sel. Top. Quantum Electron..

[15] Ellenbogen T (2009). Nonlinear generation and manipulation of Airy beams. Nat. Photonics.

[16] Shapira A, Juwiler I, Arie A (2011). Nonlinear computer-generated holograms. Opt. Lett..

[17] Saltiel SM (2009). Multiorder nonlinear diffraction in frequency doubling processes. Opt. Lett..

[18] Voloch Bloch N (2012). Twisting light by nonlinear photonic crystals. Phys. Rev. Lett..

[19] Shapira A (2012). Two-dimensional nonlinear beam shaping. Opt. Lett..

[20] Wei DZ (2019). Efficient nonlinear beam shaping in three-dimensional lithium niobate nonlinear photonic crystals. Nat. Commun..

[21] Liu S (2019). Nonlinear wavefront shaping with optically induced three-dimensional nonlinear photonic crystals. Nat. Commun..

[22] Chen PC (2021). Quasi-phase-matching-division multiplexing holography in a three-dimensional nonlinear photonic crystal. Light.: Sci. Appl..

[23] Rozenberg, E. et al. Inverse design of quantum holograms in three-dimensional nonlinear photonic crystals, Conference on Lasers and Electro-Optics, OSA Technical Digest (Optical Society of America, 2021), paper FM1N.7. 10.1364/CLEO_QELS.2021.FM1N.7.

[24] Trajtenberg-Mills S, Juwiler I, Arie A (2015). On-axis shaping of second-harmonic beams. Laser Photonics Rev..

